# Measurement and evolution of government attention to the health industry in China based on the BERTopic model

**DOI:** 10.1371/journal.pone.0329300

**Published:** 2025-08-07

**Authors:** Jian Jin, Hongbin Du, Zhaoyu Liu

**Affiliations:** 1 School of Economics, Hebei University, Baoding, China; 2 Department of Finance, Affiliated Hospital of Hebei University, Baoding, China; Zhejiang Gongshang University, CHINA

## Abstract

**Objectives:**

This paper aims to measure the government’s attention to the health industry accurately, which is crucial for understanding policy directions and resource allocation strategies.

**Methods:**

Addressing the limitations of traditional word frequency methods, such as restricted word segmentation and ambiguous terms, the BERTopic (Bidirectional Encoder Representations from Transformers Topic Modeling) is applied to measure government attention at the sentence level. Rule matching in an ambiguous dictionary, which is expanded by utilizing the word2vec model, is to achieve accurate identification of unclassified topics. This approach reveals policy concerns at the semantic level.

**Results:**

The BERTopic model is a more precise instrument for evaluating the health industry’s attention. Furthermore, significant regional differences are detected. Attention in the Northeast has declined, remained stable in the Central and Western regions, and continuously increased in the Eastern region. The main areas of government attention in the health industry are sports and fitness, environmental governance, medical services, and healthy older individuals’ care. The mode of medical care in healthy retirement has evolved to a combination of medical care and health preservation.

**Conclusions:**

It is recommended that the balanced development of the health industry across regions be promoted based on specific local conditions. Efforts should also be made to enhance the efficiency of medical services, optimize the allocation mechanism for medical resources, establish a systematic medical treatment plan, and encourage the comprehensive and coordinated development of the health industry.

## Introduction

The health industry currently holds an essential position in the national plan due to the accelerating pace of population aging and the diversity of healthcare requirements. It emphasizes the significance of strengthening general population health and rapidly expanding the health industry in “Healthy China 2030” planning. The government’s attention to the health industry and its allocation of attention directly affects the effective arrangement of resources, the optimization of service supply, and the design of industrial policies. Thus, it is fundamental to measure the health industry’s government attention accurately, which could help the researchers comprehend policy orientation and promote the scientific development of the health industry.

Theoretically, government attention is regarded as a limited resource [1]. Jones et al. integrated it into the policy decision-making process [2], generated an attention-driven policy adoption model, and proposed that variations in government attention could affect the changes in policy focus. In Britain, the Queen’s speech is a powerful measure of attention to policy-making, which would provide a conspicuous signal to different objectives, such as parliament, governing and opposition parties, the media, and the public, representing the priorities of the core executive [3]. In the United States, the president’s speeches can signal and affect policy by delivering policy tendencies to the bureaucracy [4]. In China, the government work report can intuitively reflect the change in the government’s attention to a certain field, such as the gradual increase in attention to environmental governance [5] and public health [6]. Simultaneously, the government’s constant emphasis on medical health has contributed to the emergence of pertinent supporting policies, whose purpose is to strengthen the quality and efficiency of medical services and to cultivate in the public a lifestyle of a nutritious diet and regular physical activity [7].

On the other hand, the severity of public health emergencies [8] and the prevalence of perspectives on social media [9] might determine the extent to which the government pays attention to recent health concerns. The public appeals to the government to notice the issues in certain areas through the media [10], and then the government promulgates corresponding policies and laws to satisfy the demand of the public, which indicates that the government’s attention is limited. Measuring the government’s attention is significant. Regarding measurement approaches, some researchers employ the word frequency method to measure environmental issues in government work reports [11]. In contrast, others study the government’s attention by applying the quantity of legislature [12]. Furthermore, the text analysis method is utilized to measure government attention in planning government bills, which can be quantified by extracting relevant keywords [13]. Alternatively, the topic model is employed to evaluate the government’s attention to policy documents in the concerned field [14]. Individual behaviors might impact institutional results as policymakers concentrate their attention on policy issues [15]. Therefore, there will be studies to measure the attention of government lawmakers to the agenda through their Twitter posts [16], which is a novel approach to measuring policy attention in near real-time [17].

Furthermore, the topic model has advantages for measuring government attention due to combining the meaning of words. In recent years, LDA (Latent Dirichlet Allocation) could detect potential topics in massive texts through topic distribution [18]. BTM (Biterm Topic Model) could solve the serious sparsity problems [19], which are brought about by the lack of word frequency and context information in short texts. It can capture the topic, and build the models by the word co-occurrence patterns. The LDA topic model is not applied to text classification directly and is utilized to obtain text features. Combining convolutional neural networks with LDA features could have a better performance for classifying text vectors [20]. Pretrained language models include large amounts of knowledge in the pretrained stage. It can provide a more effective method to release the burden of expensive labeled data for supervised topic classification [21]. BERT (Bidirectional Encoder Representation from Transformers) model can extract more precisely the semantic features, and combining K-means [22] or LDA model [23] with the features can enhance the accuracy of topic classification. The BERTopic model has a better performance than the conventional topic model. It generates dense clusters by employing BERT embeddings and a class-based term frequency-inverse document frequency (c-TF-IDF) to identify topic classification [24].

In summary, there is still further space to be discovered for the method to improve the measurement of government attention in the health industry. First, the word frequency method and keyword extraction method are the principal approaches employed in most empirical studies on attention. These methods have limitations when researchers need to recognize and process semantic complexity and ambiguous language in texts. Second, the word frequency method neglects the potential correlation structure between words and the topics they represent, which is not conducive to a comprehensive analysis of the government’s attention to different fields of the health industry and the dynamic trend of change. Third, BERTopic has more advantages than the traditional topic model in identifying topic classification due to the pre-trained model word embeddings. Furthermore, semantic networks are applied to reflect the network relationship of core words in the corpus, and they can also validate the result of government attention measured by BERTopic. This study uses the BERTopic model to disassemble and analyze themes in government work reports. The goal is to create a precise and semantically rich tool for measuring government attention to the health industry, which can also support empirical data and provide a theoretical foundation for health policy formulation.

## Materials and methods

### Sample

The government work reports originate from the official websites of the State Council and provincial governments. The crawler is utilized to collect the texts of the local government work report from 2003 to 2024; and the government work report of the State Council from 1993 to 2024. Due to the availability of the data, a total of 714 government work reports from the State Council, 31 provinces, autonomous regions, and municipalities across the country have been compiled.

### Measurement of government attention based on the BERTopic model

BERTopic model is a topic modeling technology based on a deep learning algorithm [25], which combines the word embedding of the Transformer model with the cluster-based word frequency statistics algorithm c-TF-IDF. Compared with LDA and DTM (Dynamic Topic Model) models, the accuracy of topic division has been ameliorated to a certain extent [26]. The BERT word embedding, UMAP dimension reduction [27], and HDBSCAN density clustering algorithm [28] are the three essential sections of the modeling procedures. To minimize the number of topics and integrate subjects that are associated, the similarity between subject phrases is finally calculated by employing c-TF-IDF. The RoBERTa_wwm model is applied for word embedding, which combines RoBERTa and whole word masking (WWM). It improves dynamic masking and replaces next sentence prediction with the full-sentence task [29].

**Fig 1:**
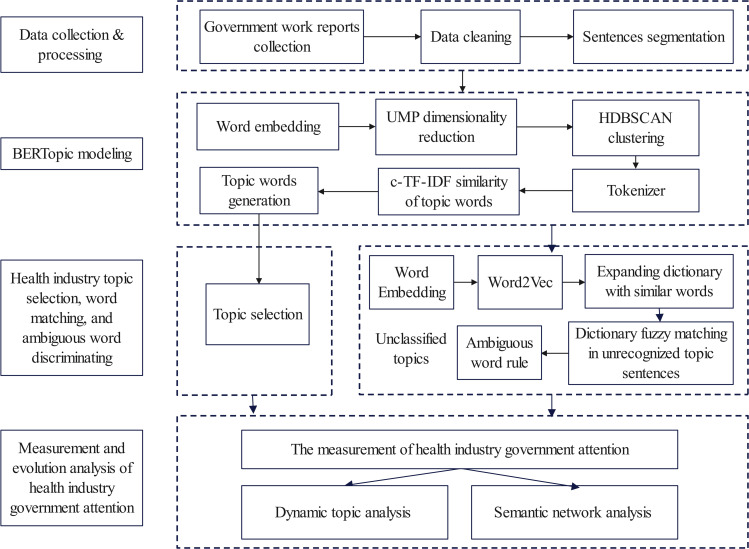
Government attention analysis flowchart.

The government’s attention to the health industry is measured by the percentage of sentences in the government work report that are linked to the industry overall. The flowchart for analyzing government attention is displayed in Fig 1, and the measuring steps are as follows:

(1) Identify the health industry-related topic sentences in the government work report text by employing the BERTopic model;
(2) Determine the number of sentences related to the health industry by selecting related topics;
(3) Apply health industry dictionary matching and ambiguous word rules to detect the health industry topic sentences in the unrecognized sentences.

All the sentences are obtained by separating the text with semicolons and periods. The sentences that are less than 15 words are removed because most of them are subtitles that contain the repeated meanings and prefixes of speech. Then, a topic model is constructed based on 259,175 sentences. The National Bureau of Statistics published a Statistical Classification of the health industry in 2019. The first level category contains medical services, health environment management and scientific research services, health talent education and health knowledge popularization, health promotion service, health security and financial services, health technology services, medicines and health products distribution services, and pharmaceutical manufacturing, etc. 25 topics related to the health industry are selected among the 299 topics, among which topic 26 contains biomedicine related sentences, as shown in Table 1. National fitness for improving health in topic 16, and pre-pregnancy physical examination in topics 49 and 231 are related to the health industry. The topic classification of some sentences might be lost due to the c-TF-IDF calculation, which is marked as −1. The missing health industry sentences are refiltered by constructing the health industry dictionary and setting ambiguous word rules, and the dictionary is the same as the one employing word frequency statistics.

**Table 1:** The category of health industry topic.

Category	Topic	Topic Word
Medical service	8	Medical health, public hospital, community health, health center, medicine
	49	Family planning, population, fertility, birth, population quality
	79	Epidemic, prevention, SARS, rebound, COVID-19
	94	Public health, epidemic, disease, treatment, AIDS
	177	Health, career, sports, education, culture
	231	Screening, free, maternal, breast and cervical cancer, death rate
Health security	13	Endowment insurance, pension, retirement, unemployment, employment injury insurance
	57	Handicapped, women and children, left-behind, care, rehabilitation
	84	Medical insurance, urban residents, serious illness, new rural cooperative medical care system, subsidy
	241	Cooperative medical care, new cooperative, pilot county, peasant, institution
	290	Settlement, hospitalize, allopatric, medical insurance, clinic
	296	Cooperative medical care, new rural, subsidy, financing, reimbursement
Sports and fitness	16	National fitness, sports, competition, sports games, Olympic games
Biomedicine	26	Equipment, materials, new energy, electronic information, biomedicine
Environmental governance	43	Air pollution, air quality, coal, pollution, dust
	48	Water quality, watershed, water body, black and odorous, water pollution control
	70	Garbage, sewage, sewage disposal, treatment rate, harmless
	150	Soil pollution, pollution, non-point sources, heavy metals, prevention
	211	Supervise, rectify and reform, feedback, environmental protection, ecology
	216	Oxygen demand, sulfur dioxide, chemistry, emissions, reduction
	275	Yellow river basin, restoration, ecology, conservation, wetland
Food and medicine safety	58	Food and medicine, safety supervision, food safety, medicine, food
Health retirement	68	Retirement, home, older individuals, bed, community
Chinese traditional medicine	186	Traditional Chinese medicine, inheritance, medicine, traditional Chinese and western medicine, clinic
	208	Chinese medicine, biomedicine, pharmaceutical industry, medical instruments, Chinese medicine materials

According to the results of the BERTopic model, 77,012 sentences are discovered to be unclassified, labeled as class −1. To precisely determine topics connected to the health industry, fuzzy matching and additional discrimination on sentences including potentially ambiguous terms like “health”, “environment”, and “ecology” need to be accomplished with the dictionary, as displayed in Table 2. 1,303 ambiguous sentences include the word “environment”, such as “international environment” and “business environment,” which accounted for around 28.11% of the 4,636 sentences; 325 (25.98%) of the 1,251 statements applying the word “health” are unidentified, including “healthy development” and “healthy and orderly development”; 194 sentences (4.47%) of the 4,341 statements employing the word “ecology” included terms like “innovation ecology” and “political ecology” that are not associated with the health industry.

**Table 2:** Ambiguous word of the health industry.

Ambiguous Word	Word list
Ecology	Innovation ecology, political ecology, poverty alleviation ecology, cultural ecology, industrial ecology
Health	Healthy development, healthy and orderly development, healthy and standardized development
Environment	International environment, complicated and mutable environment, business environment, new business environment, research environment, market environment, employment environment, investment environment, construction environment, development environment, network environment, economic environment, financial ecological environment, environmental gradient, development environment, proper environment, environmental conditions, institutional environment, challenging environment, optimization environment, domestic and foreign environment, Consumer environment, environment-friendly, social environment, external environment, facing environment, reform and innovation environment, working environment, comprehensive environment, walking environment, international communication environment, language environment, urban and rural environment, macro environment, competitive environment, entrepreneurial environment, financial environment, international and domestic environment, domestic environment, service environment, innovation environment

In the parameters of the UMAP model, n_neighbors is set to 25, n_components is set to 10, min_dist is set to 0 for the literal distance between the control data points, and cosine is utilized as the measurement standard. The HDBSCAN model employs Euclidean distance, the displayed theme words top_n_words are set to 30 in the parameter of the BERTopic model.

### Measurement of government attention based on word frequency

By referring to the “Statistical Classification of Health Industry (2019)” introduced by the National Bureau of Statistics, the fundamental words such as public health, retirement, fitness, medical care, medical reform, medicine, doctors, medical care, and other related words are extracted from the classification standard and work report as seed words. 304 keywords as a dictionary could be generated by employing the Word2vec algorithm to expand the dictionary based on seed words. The rule dictionary is implemented with both word frequency statistics and rule matching; however, the matching patterns of these two methods are different. Word frequency statistics necessitate word segmentation and then precise matching to evaluate government attention, which is calculated by the word frequency percentage of the health industry, while rule matching is to identify the rule words in sentences without limitation to word segmentation.

## Results

### The performance of BERTopic government attention

The effectiveness and precision of policy formulation are significant considerations. The BERTopic model, based on deep learning, can deal with the semantic complexity and ambiguity of text more precisely than the word frequency method. The original measurement unit of the word frequency method is a word, which is different from the metric of topic sentences. Therefore, the word frequency method needs to calculate the sentence proportion with dictionary words to compare the performance with the BERTopic method in the same dimension, which is represented by sentence segmentation and dictionary matching. In addition, more pre-trained models for word embedding are tried. The Top2Vec topic model is employed as a base model to highlight the advantage of the BERTopic model. Top2Vec produces semantic embedding by neutral networks, and it combines word2vec and doc2vec to detect the semantic structure. The performance for word embedding of “Chinese-roberta-wwm-ext” in the BERTopic model is better than “bert-base-Chinese” and “paraphrase-multilingual-MiniLM-L12-v2”, which represents as RoBERTa_wwm, Bert_base, and Paraphrase-Multilingual in Table 3. We randomly selected 1% of the samples (2,591) as the test set for manual annotation three times for validation and removed unclassified samples to compare the performance of Top2Vec and three types of word embedding in BERTopic (S7–S11 Files in S2 Data). Accuracy and precision are utilized to evaluate the performance of models. Identifying correctly the other topics except for the health industry might make the numeric value of accuracy higher. Precision could reflect the performance appropriately because the selected topic samples have a smaller percentage in the whole corpus. As displayed in Table 3, the mean accuracy of Top2Vec, Bert_base, Paraphrase-Multilingual, and RoBERTa_wwm are similar except for the unclassified samples, which are 96.32%, 96.08%, 96.89%, and 98.19%, respectively. However, there are significant differences in precision, and the BERTopic model with RoBERTa_wwm has a higher precision, which is 83.04%, and is applied in the measurement of government attention. The mean accuracies of health industry dictionary matching and the BERTopic model with RoBERTa_wwm are 91.37% and 96.56% in the whole sample, respectively. In addition, the mean precisions of dictionary matching and the BERTopic with RoBERTa_wwm are 51.29% and 74.16%. The BERTopic with dictionary matching ambiguous rule has a smaller standard deviation. It is illustrated that the BERTopic model has a more precise performance, and the approach that combines rule matching and ambiguous words could ameliorate the accuracy.

**Table 3:** The comparison of government attention’s measurement methods.

Methods	Word Embedding	Accuracy	Precision	Mean & Std Accuracy	Mean & Std Precision
Top2Vec	Doc2Vec	96.37%	72.64%	96.32% & 0.1003	72.85% & 0.3793
		96.18%	73.39%		
		96.41%	72.54%		
BERTopic	Bert_base	96.14%	72.93%	96.08% & 0.1281	73.17% & 0.2299
		95.91%	73.10%		
		96.21%	73.48%		
BERTopic	Paraphrase-Multilingual	96.83%	76.67%	96.89% & 0.0634	76.85% & 0.4511
		96.98%	77.47%		
		96.87%	76.41%		
BERTopic	RoBERTa_wwm	98.34%	83.06%	98.19% & 0.1497	83.04% & 0.7349
		98.26%	83.93%		
		97.99%	82.13%		
BERTopic & Dictionary matching Ambiguous rule	RoBERTa_wwm	96.68%	74.28%	96.56% & 0.1376	74.16% & 0.4775
		96.64%	74.67%		
		96.37%	73.52%		
Dictionary matching		91.78%	51.16%	91.37% & 0.4522	51.29% & 0.6357
		91.59%	52.13%		
		90.74%	50.59%		

### Evolution analysis of the health industry government attention

**Fig 2:**
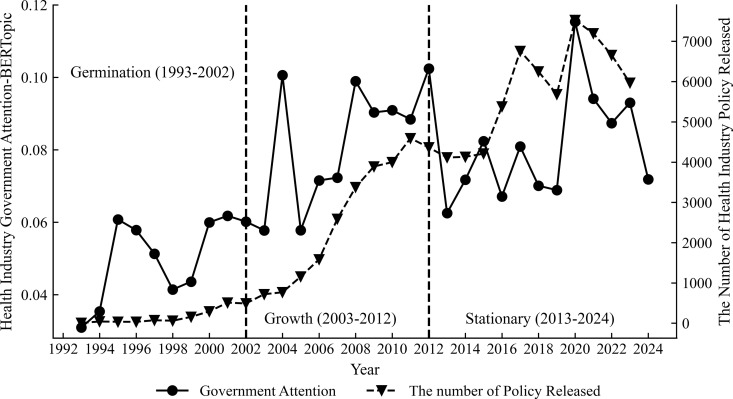
Trends of government attention and policy issuance in the health industry of the State Council from 1993 to 2024.

Fig 2 presents the evolutional trend of the central government’s attention to the health industry from 1998 to 2024 by applying the BERTopic model. During this period, it demonstrates a consistently accelerating tendency, which can be divided into three stages: the germination period (1998–2002), the growth period (2003–2012), and the stationary period (2013–2024). From 1998 to 2002, there was a preliminary upward trend in the government’s concentration on the health industry, which indicates a gradual improvement in the industry’s position about the distribution of national resources. The government’s focus on the health industry expanded dramatically between 2003 and 2012, indicating that this industry has emerged as an essential national investment and resource optimization. The attention has declined and fluctuated, but it has increased once more after 2020, which is consistent with the strategic shift of China’s health industry policy from expansion to refinement adjustment and then to refocus on the health industry in the post-epidemic era.

The search words that are extracted from “Statistical Classification of the Health Industry (2019)”, such as medical care, fitness, pollution, health insurance, medicine, doctor, medical education, medical-education collaboration, nutrition, healthy aging, drug safety, and health industry, are applied to the search from Beida law treasure platform, official websites of the provincial governments, and policy texts before December 2024 were collected through python crawler. The data illustrates that the number of policy releases has been increasing since 2000. The quantity of policy announcements can reflect the government’s attention to the health industry. Compared with the calculation of the government’s attention, these two display a similar trend.

### Evolution analysis of provincial government attention

In terms of provincial government attention analysis, compared with the word frequency method, the topic model can more precisely capture the focus of attention and changes in the provincial health industry’s government attention in different periods, which could emphasize regional differences. Therefore, it is beneficial to guide and optimize the policy formulation and resource allocation strategies of governments at all levels for the health industry.

**Fig 3:**
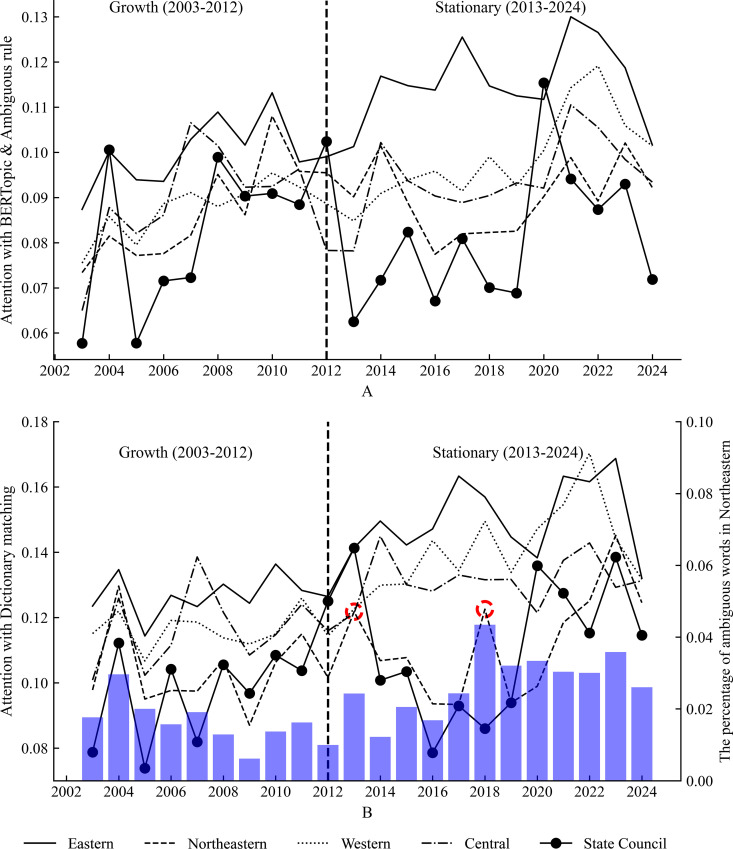
Trends of health industry government attention in four major economic regions from 2003 to 2024 based on the comparison of two methods. **(A)** Government attention is measured by BERTopic and ambiguous rule. **(B)** Government attention is measured by the word frequency method. The red circles labeled in 2013 and 2018 could highlight the misjudged effect of word frequency. The red parts correspond to the fluctuation of the bar plot, which represents the percentage of ambiguous words in Northeastern.

Four major economic regions are classified according to the policy published by the State Council, as shown in Table 4. The government attention of 31 provinces is measured due to data availability without Hong Kong, Macao, and Taiwan. The government attention of State Council in the health industry increased from 0.06 to 0.1 during 2003–2012, and decreased from 0.1 to 0.071 during 2013–2024, as shown in Fig 3. Moreover, there are significant differences among regions. From 2003 to 2012, the local government’s attention to the health industry rose simultaneously, reflecting the strengthening of China’s health industry policy system and the expansion of resource input. Nevertheless, there was a substantial divergence in different regions’ attention after 2013, and Northeastern government attention progressively decreased, with no apparent recovery until 2020. The central and western regions are fluctuating, while the eastern region displays a stable trend of continuous growth.

**Table 4:** The category of four major economic regions.

Region	Province
Eastern	Beijing, Tianjin, Hebei, Shanghai, Jiangsu, Zhejiang, Fujian, Shandong, Guangdong, Hainan, Hong Kong, Macao, Taiwan
Northeastern	Liaoning, Jilin, Heilongjiang
Western	Inner Mongolia Autonomous Region, Guangxi Zhuang Autonomous Region, Chongqing, Sichuan, Guizhou, Yunnan, Shaanxi, Gansu, Qinghai, Ningxia Hui Autonomous Region, Xizang Autonomous Region, Xinjiang Uygur Autonomous Region
Central	Shanxi, Anhui, Jiangxi, Henan, Hubei, Hunan

Fig 3 illustrates two trends depicted by the BERTopic model with ambiguous rule and word frequency method. There are two distinct bulges, and they are labeled as red circles in 2013 and 2018 in Fig 3B part. The ambiguous word frequency is calculated and displayed as a bar plot, which demonstrates a similar change with a circle labeled. This might highlight the misjudged effect of word frequency, and display the advantage of BERTopic by the comparison of two methods.

**Fig 4:**
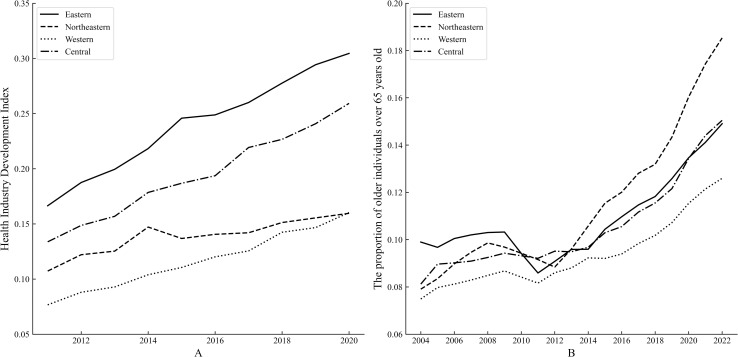
Trends of health industry development index and the proportion of older individuals over 65 years old in four major economic regions.

The health industry involves a wide range, and a single indicator cannot explain the situation of the health industry. Therefore, the entropy method is employed to compose the health industry development index. The data composed of the health industry development index is derived from the China Statistical Yearbook and the China Health Statistics Yearbook for 2011–2020, and the index system can be found in the supplement files (S5 File in S2 Data). Limited by data availability, some missing data are filled up by the moving average method. The trend of the health industry development index is similar to government attention’s trend, and the index of Northeast in 2014 displays an obvious inflection point, corresponding to the curve trend of government attention, displayed in Fig 4, which also began to decline this year. Therefore, government attention might affect the resource allocation and the development of the health industry. Moreover, the proportion of older individuals over 65 years old is extracted from the China Statistical Yearbook for 2004–2022 (S6 File in S2 Data), and the proportion in the northeast is constantly increased after 2011. The demand for health should be increasing, therefore, the government’s attention to balanced development is a principal element to improve the level of development and an important measure to meet the needs of residents.

Further analysis of government attention to the health industry in Northeast China reveals that the quantity of topic sentences pertaining to the health industry is still rising, but the proportion is shrinking, indicating that attention to other fields is growing faster and occupying a greater proportion. The sentences in topic 45 increased notably from 2013 to 2024 compared to the last ten years, which contains keywords from emerging technologies like digital, data, internet, and artificial intelligence, reflecting the promotion of digital transformation. Northeastern China has increased its focus on the data industry, digital economy, and Internet-related sectors. While this shift may not directly benefit the health industry in the region, it could lay the groundwork for future technology-driven transformations and the strategic development of smart medical care. The State Council has reduced the attention of the health industry and transferred it to other topics such as tax reduction, and enterprise transaction costs, demonstrating a priority on business environment optimization, lightening enterprise burden, and substantial economic promotion at the national level. These policies might indirectly affect the efficiency and structural optimization of resource allocation in the health industry.

### Dynamic topic analysis of health industry government attention

**Fig 5:**
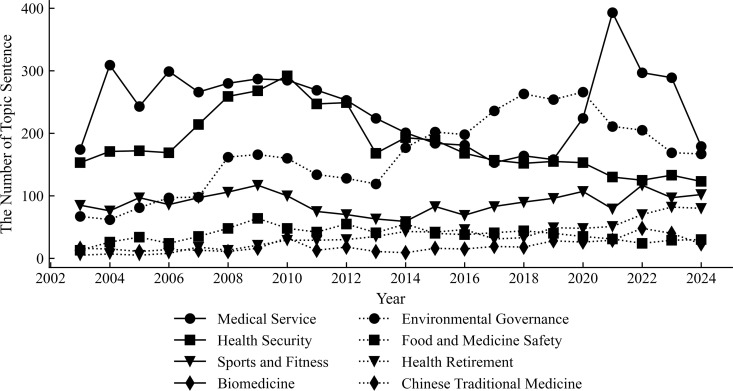
Trends of dynamic topic word frequency in the health industry from 2003 to 2024.

Topics_over_time function of the bertopic package in Python is applied to generate a time series dynamic topic model of government attention in the health industry. The model indicates that an extensive portion of the government work report contributes to the four topics of medical services, health security, environmental governance, and sports and fitness, as displayed in Fig 5. The government work reports in 2004 and 2021 illustrate an enormous rise in the quantity of sentences associated with medical service topics due to the SARS epidemic in 2003 and the COVID-19 epidemic in 2020. This reflects the impact of public health events on the demand for medical resources and the government’s emphasis on medical services during crisis response. From 2003 to 2012, fundamental social security systems, such as health insurance and pension insurance, were building and reinforcing gradually, during this time, the attention they acquired was higher than in the years after 2012. The sentence percentage mentioning health security in the work report has declined as the social security system has developed and subsidy standards have improved. This indicates that China is making incremental progress towards optimizing the industry structure and enhancing the effectiveness of resource allocation to the health industry. The environmental governance topic encompasses environmental pollution control and environmental monitoring such as soil pollution, water pollution, air pollution, and garbage disposal with higher attention, which indicates the government’s investment in environmental protection and public health in different stages. Sports and fitness topic has maintained high attention; nevertheless, the frequency of healthy retirement has steadily increased since 2016 and will be close to sports and fitness after 2021, with a more enhancive public’s demand for health preservation and perception of the meaning of medical care.

**Fig 6:**
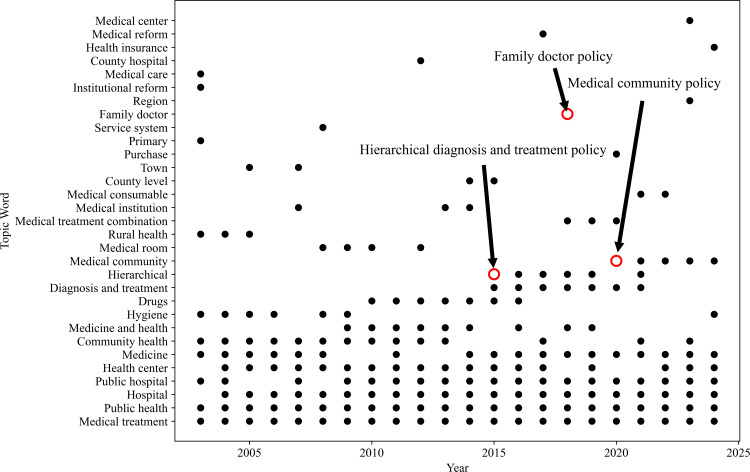
High-weighted topic words’ occurrence scatter plot in topic 8. The red circle represents the high-weighted words that occur the first time with the publication of a new policy, and the words could continue to appear for several years based on the importance of the policy. For example, hierarchical diagnosis and treatment policy appears from 2016 to 2019 in topic 8.

The Top 10 keywords for each topic are extracted by employing the weight of c-TF-IDF as the selection criterion for keywords. The topic words about medical infrastructure construction, medical system reform, cooperative medical care system, community health service system construction, medical insurance policy improvement, and drug management represent government issues that demonstrate the essential government provision of basic medical services and the construction of security systems before 2015, majoring in topic 8. As shown in Fig 6, the weight of “hierarchical” and “diagnosis” enlarged from 2015 to 2016, which is closely related to guidelines on promoting the construction of hierarchical diagnosis and treatment systems released by the State Council. This indicates that the guidance of policies transfers to optimize the structure of medical services and strategically distribute medical resources, boosting the efficiency and equity of the medical service system. In 2018, the increasing weight of the topic word “family doctor” illustrates the concentration on the innovation of the primary medical care model. It aims to strengthen the capacity of primary health services, reduce medical expenditures, and facilitate the implementation of preventive health policies through the advancement of family doctor contractual services. The transformation is crucial for enhancing the quality of medical care for residents, decreasing the expense of medical care for the general public, and progressing the principles of health management and preventative healthcare. The continuous occurrence of the topic word “epidemic” in 2020 indicates the significant influence of public health emergencies. The construction of regional medical centers and the balanced distribution of medical resources have become new policy orientations with the emergence of the “medical community”. The development objective of local medical services has switched to concentrating on more effectively integrating and employing medical resources, enhancing collaboration on resources, and establishing collaborative services within the community. The government’s attention to the medical services topic has gradually stabilized with progressive policy implementation.

**Fig 7:**
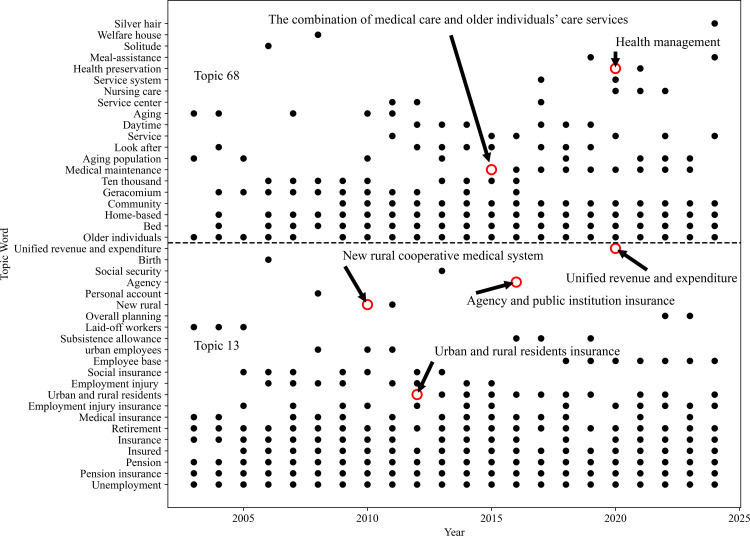
High-weighted topic words’ occurrence scatter plot in topics 13 & 68. The red circle represents the high-weighted words that occur the first time with the publication of a new policy, and the words could continue to appear for several years based on the importance of the policy. For example, urban and rural residents’ insurance policy appears from 2012 to 2019 in topic 13, and the combination of medical care and older individuals’ care services policy appears from 2016 to 2022 in topic 68.

In the health security topic, the issue mainly focuses on diversified health security services such as medical insurance, work-related injury insurance, pension insurance, and social insurance, majoring in topic 13. Fig 7 demonstrates the keywords of policy implementation. The word “new rural” originated in 2010 and is closely associated with the new rural social endowment insurance system, which has been operational since 2009 on a pilot basis. This represents a breakthrough in the development of China’s urban-rural integration social security system. To strengthen resource allocation in the health industry and improve equity, the government work report stipulates explicitly that medical insurance for urban employees and urban residents and the new rural cooperative medical system should be implemented to guarantee comprehensive coverage for both urban and rural residents. In 2012, the phrase “urban and rural residents” frequently appeared, to achieve complete coverage of urban and rural residents’ pension insurance, as well as extending the scope of the new rural insurance pilot and the urban residents’ pension insurance pilot. In 2016, the words “agency” gained weight. The word “employees” in 2019 indicated that enterprise employees’ basic pension insurance fund should develop a central adjustment mechanism. In 2020, the phrases “unified revenue and expenditure” and “overall planning” received more consideration to steadily implement the national overall planning of enterprise employees’ basic pension insurance. It is exploring ways to address the problem of interregional pension imbalance and stimulate appropriate circulation and effective execution of pension insurance funds through the innovative policy strategies of the country. Simultaneously, these initiatives not only help optimize the allocation of resources in the health industry but also achieve a more equitable and sustainable health industry development strategy at the national level.

In the environmental governance topic, topic 43 is primarily concerned with reducing pollutant emissions to improve air quality, such as “coal burning” and “motor vehicles”. They have the largest percentage of air pollution. The word “pm2.5” has larger weight since 2012. “Supervision” is constantly considered in topic 211, which indicates enforcing environmental laws and regulations more strictly and punishing environmental violations severely are necessary. This is an essential initiative in reducing the risks of environmental pollution-related illnesses as well as the associated social and medical costs. The policy of promotion on the river manager system released in 2016 elevates the weight of the topic word “river manager” after 2017. The river manager system has been implemented to prevent water pollution at different levels under the Law of the People’s Republic of China on Water Pollution Prevention and Control, recently updated in 2017. Effective prevention of water pollution can directly guarantee the safety of drinking water and reduce the incidence of various diseases caused by water pollution. It is conducive to improving the efficiency of water resource utilization, saving a quantity of public health costs, and promoting the sustainable development of the economy.

In the healthy retirement topic, the topic word “facilities” demonstrates that the construction of infrastructure for healthy older individuals’ services has been emphasized by work reports since 2005. It could strengthen and ameliorate the healthy older individuals’ services system by increasing the supply of public service facilities and beds. The investment at this stage responds to the changes in social demand brought about by the aging population, and it symbolizes the government’s preference for allocating resources from the health industry to older individuals’ health services. Tracking back to the sentences including the frequent topic words “retirement”, “home”, and “community”, which have increasing weights, an evolving policy from a centralized retirement pattern to a more diversified and more practical retirement pattern is represented. Simultaneously, social resources are encouraged to participate in community-based and home-based older individuals’ care services, aiming to optimize the distribution of medical service resources and upgrade the quality of life and health conditions of the older individual population. As shown in Fig 7, since 2016, the Guiding Opinion on Promoting the Combination of Medical Care and Older Individuals’ Care Services was released, with the increasing weight of the topic word “medical care with retirement”. This illustrates that the strategy of combining medical care and retirement services is officially confirmed, and the government’s attention is transforming from the older individuals’ services only to the combination of medical care and older individuals’ services. It is to effectively reduce the disease burden of the older individual population and the intensity of medical service utilization. Simultaneously, healthy economic benefits would be improved by saving public health expenditures. In 2020, the appearance of the topic word “health preservation” led to the further expansion of a multifaceted serving network, including health management and health care. Early health intervention and continuous health management could prevent the emergence and progression of chronic diseases and reduce medical pressure during the aging process. Health preservation would promote the economic growth of the health industry through industrial integration and development. The evolution path of topic words is as follows: the construction of older individuals’ care service facilities → home care → the combination of medical care and older individuals’ care services → the combination of medical care and health preservation.

### Semantic network analysis of government attention in the health industry

The dynamic topic analysis by BERTopic could detect the trend of topic changing, however, the semantic network can explore the relationship among the words more specifically to gain insight into the evolution of the government’s focus on the health industry. The corpus that the semantic network applies is based on the health industry related texts that are identified by the BERTopic model. It is constructed by word segmentation with the health industry dictionary, and the co-occurrence relationship among the top 100 most frequent words was determined by the PMI to further explore the semantic correlation between words. The significance of the connection between two words was weighted by the value of PMI.

Pointwise Mutual Information (PMI) is a statistical measurement employed to evaluate the correlation between two words or events that occur simultaneously. The correlation between word pairs in the government work report corpus is calculated by PMI, and the semantic network is constructed using the correlation as the edge of the node. The Formula 1 is as follows:


$$PMI(x,y)=log\frac{P(x,y)}{P(x)P(y)}$$ (1)

*P* (*x, y*) represents the probability that the word *x* and the word *y* appear in the text simultaneously. *P* (*x*) and *P* (*y*) represent the probability that the word *x* and the word *y* appear in the entire corpus, respectively. The correlation between two words *x* and *y* can be represented by the PMI value. When the PMI value is greater than 0, it indicates that *x* and *y* are positively correlated; The larger the PMI, the stronger the correlation between *x* and *y*. If PMI is equal to 0, it illustrates that *x* and *y* are independent.

**Fig 8:**
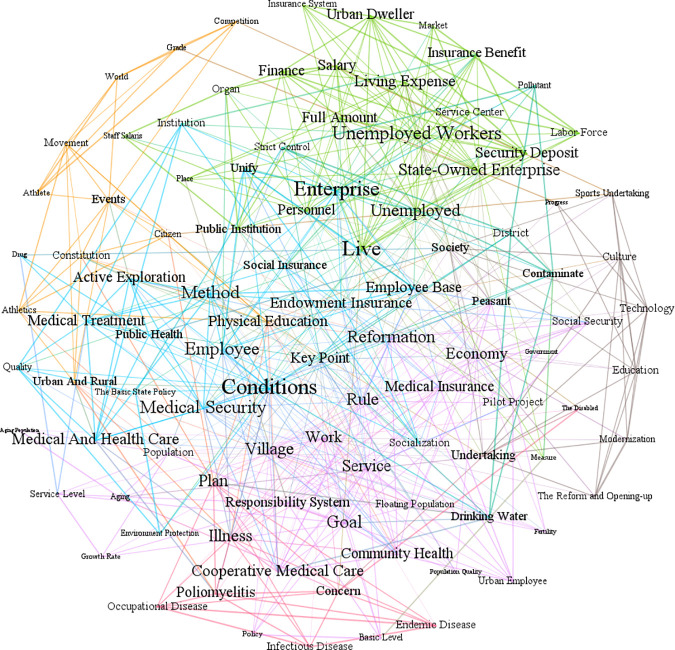
Semantic co-occurrence network of the State Council’s government attention in the health industry from 1993 to 2002.

In the germination period (1993–2002), the government’s attention to the health industry was initially increased; however, the growth of attention was relatively small. At this stage, the country focuses more on economic development, deepening economic structural reform, and expanding its opening up to the outside world, and the government’s attention to the health industry fluctuates between 0.031 and 0.061. According to the statistics of the topic sentences in the health industry, there are 3,891 sentences in the government work report of the State Council, of which 222 are related to the health industry, accounting for 5.71%. During this period, Gephi is applied to calculate the average weighted degree to indicate the weight of each node, which is represented by the font size of keywords in the Frutchterman Reingold layout. Subsequently, the semantic co-occurrence network of the report is clustered according to 7 divided modules by community structure. Nodes are more closely connected within the same community than with other communities. 7 modules can be explained as rural health, medical insurance, security system, sports undertaking, state-owned enterprise, and pollutant control according to the keywords and color. Fig 8 illustrates that, in the context of an aging population, a comparatively considerable proportion of terms for urban residents are related to social security and medical insurance. The primary objective is to guarantee essential living and medical security for both urban and rural residents, and the establishment of personal accounts has received consideration. In the medical and health module, the appearance of “active exploration” can additionally reflect the initial stage of exploring medical security methods and sports management system reform. Regarding physical fitness, sports competitions, and physical activities have been promoted to improve the national physique and health. The modules related to food and drugs and the environment are relatively small, and more emphasis is placed on the supervision and management of the environment, food and drugs, and public health.

In this paper, the intermediate centrality and eigenvector centrality of semantic co-occurrence networks is calculated in Gephi to determine the emphasized keywords. A word with a high degree of betweenness centrality will constantly appear on the shortest path between other word pairs, which indicates that the word may serve as a “bridge” in the network, connecting other conceptual groups or topics. Eigencentrality might demonstrate that a phrase has a high frequency of occurrence and an established association with other essential terms, which can partially demonstrate the primary goals or significant influencing aspects of the policy. The top 10 keywords of centrality were selected, as shown in Table 5, reflecting the focus on accelerating the reform of social security systems such as basic pension insurance, medical insurance, and unemployment insurance, ensuring the minimum living security of unemployed workers, and promoting the urban medical insurance system and rural cooperative medical care system.

**Table 5:** Keywords’ centrality of State Council in health industry semantic network from 1993 to 2002.

Keywords	Betweenness centrality	Keywords	Eigencentrality
Work	947.7070	Village	1.0000
Live	417.3649	Work	0.9819
Reformation	329.7384	Service	0.9576
Economy	304.1644	Rule	0.9307
Conditions	278.4445	Conditions	0.8959
Enterprise	276.1710	Economy	0.8608
Key Point	253.7990	Reformation	0.8531
Service	213.4649	Employee	0.7605
Physical Education	202.2840	Goal	0.7574
Events	189.4053	Medical Insurance	0.7559

**Fig 9:**
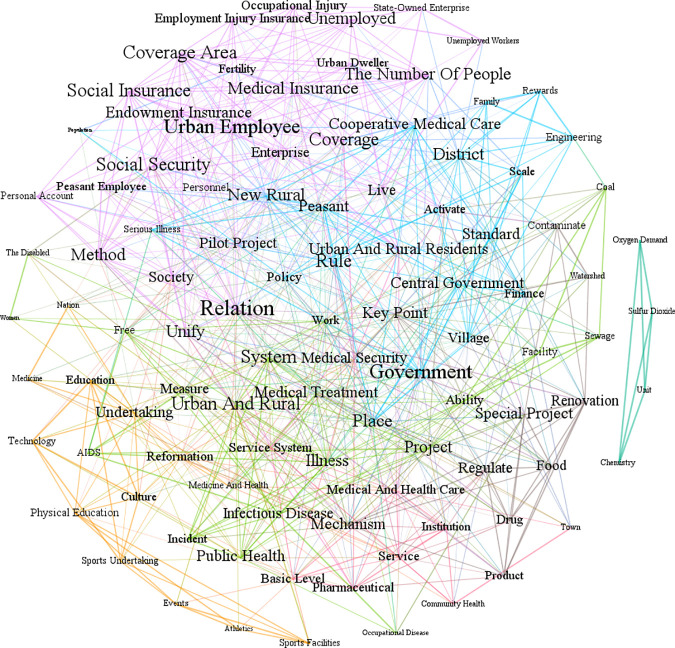
Semantic co-occurrence network of the State Council’s government attention in the health industry from 2003 to 2012.

In the growth period (2003–2012), the government attention to the health industry increased from 0.05 to 0.1, with a large growth rate. Considering the topic sentences of the health industry, the government work report of the State Council is divided into 4797 sentences, and 501 sentences are related to the health industry, accounting for 10.44%, which is improved compared with the previous stage. As shown in Fig 9, there are 7 modules; however, the direction of focus has been changed. With the deterioration of environmental problems, the topic of air pollution has gained more weight, and more attention has been paid to setting targets for pollutant emissions and improving environmental issues. After the SARS epidemic in 2003, there was an expansion in the severity of “infectious disease” and a focus on emergency measures. The proportion of attention paid to social security insurance, such as medical treatment, occupational injury, and maternity, is increasing. Urban workers and residents have received more consideration in pension and medical insurance, and pilot projects intended for creating individual accounts have been conducted. In terms of physical health, sports facilities have been established in urban and rural areas to provide residents with suitable opportunities and environments for fitness.

As shown in Table 6, the words that emerge from the betweenness centrality and eigencentrality are “rule”, “medical treatment”, “pilot project” and so on. There are some novel pilot projects established to promote medical rule reformation, which involves individual pension accounts for urban workers, a new type of pension insurance for rural residents, medical insurance for urban residents, a new type of rural cooperative medical care system, the reform of public hospitals, the first diagnosis in communities, free pre-pregnancy health check-ups, and free cervical and breast cancer screening and treatment for women. Regarding medical care, it primarily focuses on enhancing rural health and medical services, reorganizing public hospitals, building systems for disease prevention and control, and designing emergency programs for public health and medical treatment.

**Table 6:** Keywords’ centrality of State Council in health industry semantic network from 2003 to 2012.

Keywords	Betweenness centrality	Keywords	Eigencentrality
Urban And Rural	271.4228	Rule	1.0000
Rule	229.4114	Pilot Project	0.8116
Work	209.6402	Urban And Rural	0.7960
System	190.3672	System	0.7654
Pilot Project	178.6900	Society	0.7091
Key Point	171.9203	Medical Treatment	0.7053
Undertaking	160.2556	Government	0.6962
Medical Treatment	152.9607	Relation	0.6920
Society	149.7460	New Rural	0.6856
Village	144.0967	Urban Employee	0.6594

**Fig 10:**
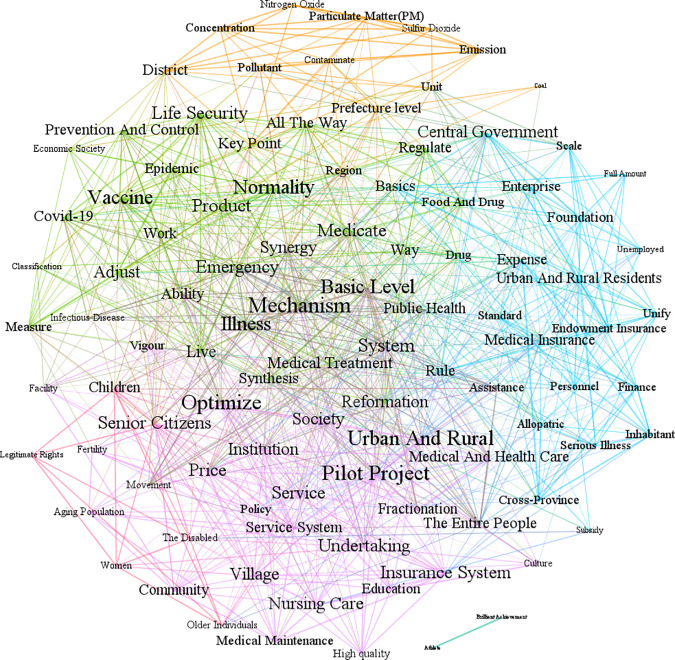
Semantic co-occurrence network of the State Council’s government attention in the health industry from 2013 to 2024.

In the stationary period (2013−2024), the occurrence of COVID-19 has given the terms like “emergency”, “normality”, “precise prevention and control”, and “vaccine” greater weight, and raised the attention to public health topics. Government attention fluctuates between 0.06 and 0.11. The government work report of the State Council is divided into 4770 sentences, and 499 sentences are related to the health industry, accounting for 10.46%, which is close to the previous stage. As displayed in Fig 10, the relationship among nursing care, medical maintenance, and insurance systems is detected at the top of the co-occurrence network. The government has become increasingly concentrated on the long-term care insurance system, which is crucial to medical maintenance, and it is progressively transitioning from providing medical treatment only to providing both medical care and medical maintenance for older individuals. The application of hierarchical diagnosis and treatment in the context of medical services is another topic of focus. Words related to air pollution continue to carry more weight when discussing environmental issues, whereas words related to sports and fitness retain less weight.

As shown in Table 7, the words that appear from both betweenness centrality and eigencentrality include medical treatment, reformation, optimization, etc. It is indispensable to ameliorate the capacity of the community to prevent and treat diseases, to improve the hierarchical diagnosis and treatment system, and to promote the sinking and balanced distribution of high-quality medical resources. In terms of optimization, it mainly involves optimizing the supply of older individuals’ care services, fertility policies, epidemic prevention and control measures, and promoting pilot projects for long-term care and hierarchical diagnosis and treatment. There are several aspects related to reformation, which include public hospitals, drug markup policies, the linkage of medical insurance, medical care, and medicine, the new drug review system, the medical device review system, the hierarchical diagnosis and treatment system, the pension insurance system, food and drug supervision, and the disease supervision system.

**Table 7:** Keywords’ centrality of State Council in health industry semantic network from 2013 to 2024.

Keywords	Betweenness centrality	Keywords	Eigencentrality
Key Point	262.4839	Reformation	1.0000
Service	230.8230	Service	0.9670
Reformation	183.5845	Rule	0.9065
Rule	178.5696	Pilot Project	0.8945
Standard	153.5976	Medical Treatment	0.8711
Medical Treatment	140.6170	System	0.8452
Society	134.5161	Mechanism	0.8078
Optimize	121.7056	Optimize	0.8018
Mechanism	109.7240	Urban And Rural	0.8011
Senior Citizens	105.2713	Basic Level	0.7870

## Discussion

Word frequency method and keyword extraction continue to be prevalent approaches for measuring government attention. For instance, keywords are retrieved from documents that the Mexican President has published about climate change to demonstrate the government’s attention and agenda change [30]. Topic models can be verified by applying the models to measure the concerning area of government attention in some studies [31]. The BERTopic model has better performance than LDA in topic extraction and is the first application in the measurement of government attention [32]. Simultaneously, BERTopic is applied to detect transitions in topics for particularly vulnerable groups and to prevent suicide by text message on the COVID-19 hotline [33]. Moreover, measuring the government’s attention by the number of words in a specific field in speeches, reports, conference questions, and other materials is feasible [34]. However, the attention and the impact are not equivalent, and providing the same attention in different places might not generate the same impact.

The BERTopic model is suitable to measure the government’s attention to the health industry through government work reports because the government report is a summary of the whole year’s essential events and a plan for next year, which contains many themes. However, the multi-classification model is not appropriate for identifying a large number of themes in health industry. The BERTopic model can discriminate the topics through text clustering, and can reduce the manual labeling cost relative to the multi-classification model. Compared with the traditional word frequency method, it represents higher accuracy and comprehensiveness in capturing the policy focus. There are two differences between these two methods. First, the accuracy of word segmentation is limited by the size of the syllabus and its update frequency. It is easy to disregard emerging words or fail to precisely identify fundamental ideas containing multiple phrases, like “hospital” in the rule dictionary. However, compound nouns like “Tiantan hospital” might not be effectively identified and accurately matched after word segmentation processing by the traditional word frequency method. Some segmented phrases cannot properly represent the meaning of the entire sentence, however, the BERTopic model identifies the sentence, which could provide a more comprehensive meaning than segmented phrases. With the advantages of the pre-training model, the BERTopic model can comprehend and extract semantic information from textual data more effectively, enabling it to recognize the primary elements of policy with higher accuracy. Second, the optimization of the topic model combined with the ambiguous word rule could compensate for the problem of the word frequency method, which contains the related words of the health industry but does not represent the precise meaning. For example, “promoting the healthy development of tourism” refers to “health”, but the principal objective is to encourage tourism. Another example is “creating a good innovation ecological environment”, where “innovation ecological environment” actually refers to the innovation environment rather than environmental governance issues. The improved topic model can avoid the misjudgments illustrated in the examples. It offers a more accurate measurement of government attention and supports the optimization of policy layout.

## Limitation

Government reports provide a summary of the entire year’s events and cover many subjects. The BERTopic model is appropriate to identify the many topics of government reports, but the percentage of unlabeled part accounts for some proportion, and combining ambiguous words rule cannot identify all the unlabeled samples precisely. Moreover, topic 16 is related to sport and fitness, but improving health through physical exercise is included in the health industry rather than competitive sports. In topic 13, unemployment insurance is outside the area of health industry, but this insurance is as important to social security as medical insurance. Future work will concentrate on trying another cluster model after word embeddings to reduce the unlabeled samples and to discover more precise and specific topic clusters.

In terms of language promotion, Chinese texts are trained without translation, which are different from the English text in the training model, such as the preference of writing policy text and the habit of speaking speeches, etc. Furthermore, due to the different writing preferences of government work reports, the length of the report might affect the percentage. The method of ambiguous words might not be applicable to all language documents. However, the BERTopic model is still an available choice for the classification of policy texts.

Health industry government attention can only detect the government agenda and discussion, which are recently considered and discussed topics but it cannot evaluate the effects of a specific field. Due to the limited space, the evolution of health industry government attention by region illustrates the priorities of each province, but cannot evaluate the effect of other fields. The breakpoint of government attention might be generated by multiple events, and the reason for government attention’s changing and the multifaced effects of government attention will be considered in the future.

## Conclusion

This paper proposes a framework for analyzing government attention by topic model and semantic network, a method to measure the health industry government attention based on the BERTopic model, and detects the dynamic change in government attention to the health industry. First, the BERTopic model combined with the rule of ambiguous words can more accurately capture the government’s focus on the health industry. Applying the RoBERTa_wwm pre-trained model for word embedding can improve the accuracy and precision of the topic classification.

Second, it is demonstrated that the health industry government attention of the State Council and local governments has transitioned from general growth to differentiated development through the evaluation of regional government attention. Before 2013, the State Council and each region had a similar trend. After 2013, the attention of the State Council and the northeast region in the health industry declined, the central and western regions remained stable, and the eastern region had a moderate expansion. It is imperative for differentiated health industry policy formation according to local conditions. Constantly strengthening the innovation capability of the eastern region to promote the transformation and upgrading of the health industry, consolidating and expanding the health industry’s achievements in the central and western regions, and modifying the northeastern region’s policies to encourage the health industry’s vitality might be the appropriate strategy in the future, which could be conducive to the balanced regional development of the health industry.

Third, the topics of medical service, health security, environmental governance, and sports and fitness have received more attention through the dynamic topic analysis of BERTopic. Since 2013, attention to health security has declined, while that to environmental governance has increased. The weight of words related to epidemics and emergencies in medical service has increased, resulting in strong volatility. Health and senior care issues received more attention after 2016. The linkage reform of medical care, medicine, and medical insurance is an essential issue in the current medical system. The reform of the medical system should be promoted continuously to generate a scientific hierarchical diagnosis and treatment service system, which could reduce people’s burden of medical treatment effectively, and ameliorate the efficiency of medical services. In public health, it is indispensable to enhance the emergency management system for public health emergencies and improve the resilience and response speed of the medical service system. The increasing attention to environmental governance illustrates the relationship between the ecological environment and health, and the prospect of green life should be propagated to promote the development of the green health industry. The construction of rural medical infrastructure should be emphasized to narrow the gap between urban and rural medical services. It is urgent to utilize technological methods sufficiently, to promote the sinking of high-quality medical resources, and to establish an orderly pattern of medical treatment.

Finally, in medical care services, the emphasis transitions from the reform of public hospitals and the construction of rural health facilities to the linkage reform of medical insurance, medical care, and medicine and the improvement of hierarchical diagnosis and treatment systems. The previous topic about the basic security of employees has been changed to the optimization of elderly care service supply, with the large weight of social security topic words. In the healthy retirement topic, based on the relationship among nursing care, medical maintenance, and the insurance system at the 2013–2024 co-occurrence network, a similar relationship with dynamic topic analysis has been acquired by the network for cross-validation, which is continuously transitioning from providing medical treatment only to the combination of medical care and health preservation.

## Supporting information

S1 Data Data and Code. This file includes all the original data utilized in the study and the corresponding code applied for data processing and analysis.
(RAR)

S2 Data Analysis Results. This file includes the health industry related indicators constructed using other data sources, the validation sample with different measurement methods, and the detailed results of tables and figures, which supplement the content of tables and figures mentioned in the main text.
(RAR)
